# Abnormal Hypermethylation at Imprinting Control Regions in Patients with S-Adenosylhomocysteine Hydrolase (AHCY) Deficiency

**DOI:** 10.1371/journal.pone.0151261

**Published:** 2016-03-14

**Authors:** Antje Motzek, Jelena Knežević, Olivier J. Switzeny, Alexis Cooper, Ivo Barić, Robert Beluzić, Kevin A. Strauss, Erik G. Puffenberger, S. Harvey Mudd, Oliver Vugrek, Ulrich Zechner

**Affiliations:** 1 Institute of Human Genetics, University Medical Center of the Johannes Gutenberg University Mainz, Mainz, Germany; 2 Institute Ruđer Bošković, Division of Molecular Medicine, Zagreb, Croatia; 3 Institute for Toxicology, University Medical Center of the Johannes Gutenberg University Mainz, Mainz, Germany; 4 Department of Pediatrics, University Hospital Center Zagreb & University of Zagreb, School of Medicine, Zagreb, Croatia; 5 Clinic for Special Children, Strasburg, Pennsylvania, United States of America; 6 Franklin and Marshall College, Lancaster, Pennsylvania, United States of America; 7 Laboratory of Molecular Biology, National Institute of Mental Health, Bethesda, Maryland, United States of America; University of Bonn, Institut of experimental hematology and transfusion medicine, GERMANY

## Abstract

S-adenosylhomocysteine hydrolase (AHCY) deficiency is a rare autosomal recessive disorder in methionine metabolism caused by mutations in the *AHCY* gene. Main characteristics are psychomotor delay including delayed myelination and myopathy (hypotonia, absent tendon reflexes etc.) from birth, mostly associated with hypermethioninaemia, elevated serum creatine kinase levels and increased genome wide DNA methylation. The prime function of AHCY is to hydrolyse and efficiently remove S-adenosylhomocysteine, the by-product of transmethylation reactions and one of the most potent methyltransferase inhibitors. In this study, we set out to more specifically characterize DNA methylation changes in blood samples from patients with AHCY deficiency. Global DNA methylation was increased in two of three analysed patients. In addition, we analysed the DNA methylation levels at differentially methylated regions (DMRs) of six imprinted genes (*MEST*, *SNRPN*, *LIT1*, *H19*, *GTL2* and *PEG3*) as well as Alu and LINE1 repetitive elements in seven patients. Three patients showed a hypermethylation in up to five imprinted gene DMRs. Abnormal methylation in Alu and LINE1 repetitive elements was not observed. We conclude that DNA hypermethylation seems to be a frequent but not a constant feature associated with AHCY deficiency that affects different genomic regions to different degrees. Thus AHCY deficiency may represent an ideal model disease for studying the molecular origins and biological consequences of DNA hypermethylation due to impaired cellular methylation status.

## Introduction

AHCY (S-adenosylhomocysteine hydrolase; EC 3.3.1.1) removes SAH (S-adenosyl-L-homocysteine) formed in each S-adenosylmethionine (SAM)-dependent methyltransferase reaction by catalysing its hydrolysis to adenosine and homocysteine [1, 2]. In eukaryotes, SAM is the main source of methyl groups used by methyltransferases to modify DNA, RNA, proteins and cellular metabolites [3].

A complete loss of AHCY leads to embryonic lethality in mice [4]. Compound heterozygous mutations of the human *AHCY* gene resulting in a reduced activity of AHCY have been reported in eight patients [5–11]. Patients displayed characteristic biochemical abnormalities with marked elevations of plasma SAH, SAM, methionine and creatine kinase accompanied by a significant decrease in the SAM/SAH ratio. Clinical characteristics include myopathy, hypotonia, psychomotor delay, abnormalities of liver function tests and delayed myelination [5–11]. Dietary methionine restriction and supplementation with creatine and phosphatidylcholine can lead to improved myelination and psychomotor development [5, 6]. However, this dietary therapy of patients did not eliminate or decrease elevated global leukocyte DNA methylation, a further important feature of AHCY deficiency that was measured by incorporation of [^3^H]dCTP into DNA of patients compared to that of control individuals [5, 6].

DNA methylation is linked to transcriptional repression via recruitment of methylcytosine-binding proteins and chromatin-modifying factors to *cis*-regulatory elements. It is crucial for normal development and differentiation, with particular importance in processes like genomic imprinting, X-chromosome inactivation, silencing of transposons and inactivation of tumor suppressor genes [12–14].

Genomic imprinting leads to a parent-of-origin-specific gene expression due to allele-specific DNA methylation. Imprinted genes are most often found in clusters and regulated by CpG (cytosine phosphate guanosine)-rich differentially methylated regions (DMRs). Methylation at these DMRs is more static, as it is established during the development of germ cells and maintained during the life cycle of the developing organism with only minor modifications [15]. Many imprinted genes have essential regulatory functions in fetal and placental growth and development, somatic differentiation, as well as neurological and behavioral processes. Imprinting defects can cause severe syndromes with physical and mental retardation, e.g. Prader-Willi syndrome and Angelman syndrome [15]. Since imprinted genes robustly maintain their DNA methylation in healthy somatic tissues, they can serve as ideal indicators for the epigenetic stability of a cell or an individual [16].

LINE1 and Alu repetitive elements are derived from retrotransposons and encompass 17% and 10% of the human genomic sequence, respectively [17]. The 6000–7000 bp long full length LINE1 element is capable of autonomous retrotransposition, but the majority of LINE1 elements represent 5′-truncated, internally rearranged or mutated sequences that are retrotransposition-defective [18]. The ~ 300 bp long non-autonomous Alu elements belong to the family of short interspersed nuclear elements (SINEs). They are CpG-rich and account for one-third of the total CpG sites in the human genome [19]. In somatic tissues, LINE1 and Alu elements usually display high methylation levels that inhibit retrotransposition activity [20]. Thus, LINE1 and Alu methylation levels are also regarded as excellent surrogate indicators of genome wide alterations in DNA methylation.

The aim of this study was to more thoroughly determine leukocyte DNA methylation changes in patients with AHCY deficiency by quantifying global DNA methylation and DNA methylation at DMRs of six imprinted genes (*MEST*, *SNRPN*, *LIT1*, *H19*, *GTL2* and *PEG3*) as well as Alu and LINE1 repetitive elements.

## Results

### Global DNA methylation analysis

**Fig 1:**
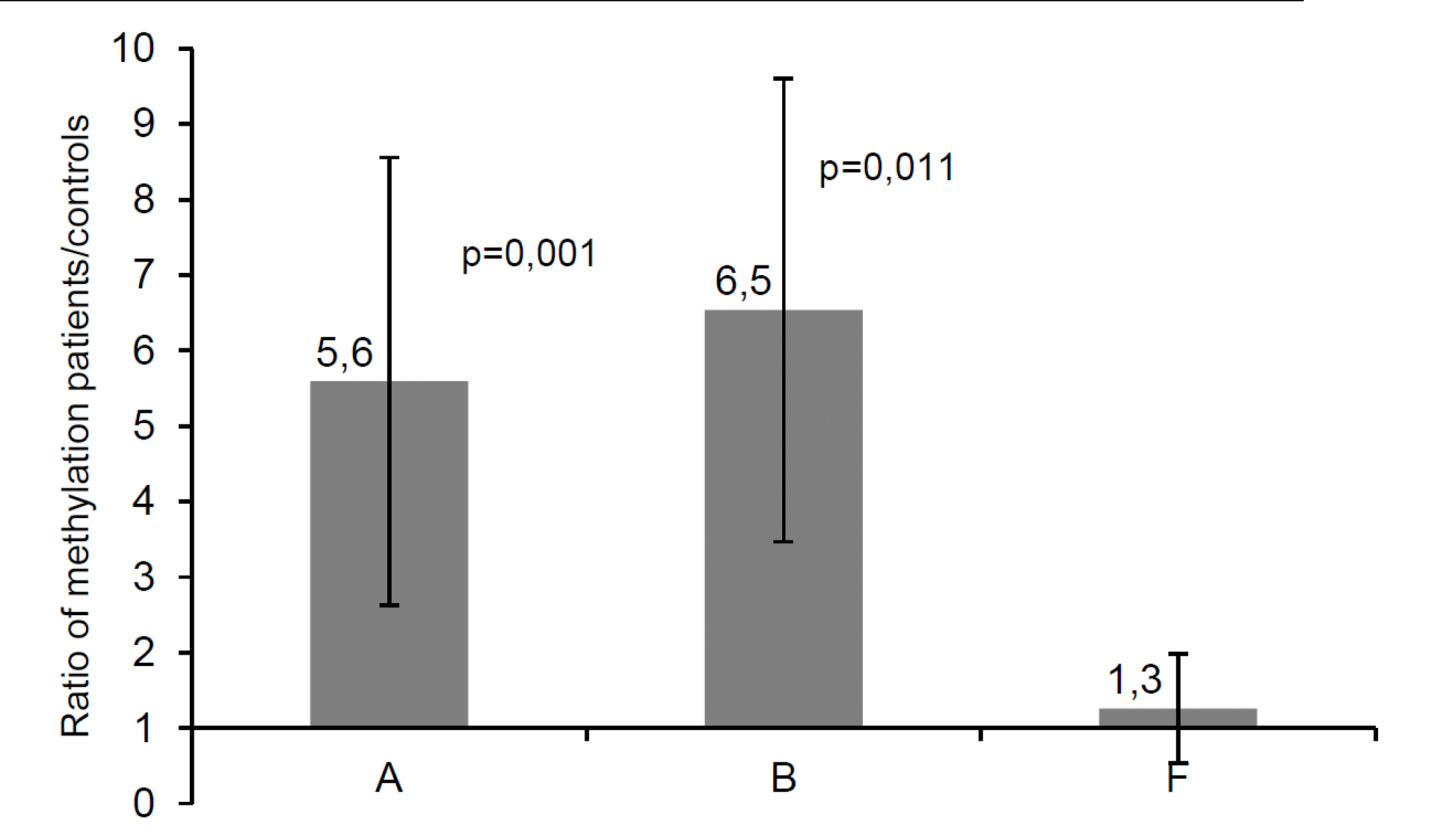
Genome wide methylation levels in three patients with AHCY deficiency and controls. Patients A and B showed genome wide methylation levels more than five times as high as those of controls, patient F displayed a genome wide methylation level similar to that of controls. The bars display the ratio of methylation levels of the three patients normalised to that of three control samples in three independent measurements. Variation between the three control samples was included via error propagation.

We measured global DNA methylation levels in patients A, B and F (Table 1) using the MethylFlash Methylated DNA Quantification Kit (Epigentek). Patients A and B showed methylation levels that were 5.6 ± 3.0 times (patient A, p = 0,001) and 6.5 ± 3.1 times (patient B, p = 0,011) as high as those of the controls (n = 3) (Fig 1). In contrast, patient F displayed a methylation level similar to that of controls (1.3 ± 0.7 times, Fig 1). To confirm that the three control samples analysed in parallel to the three patient samples are representative for the normal population, 10 additional age- and sex-matched controls (5 females and 5 males) were analysed in a subsequent experiment together with the DNA of patient B. This experiment also detected a markedly elevated methylation level of patient B compared to the 10 control samples. These control samples displayed methylation levels very similar to those of the three controls of the first experiment and no significant sex difference in methylation levels (S1 Fig). Due to the limited amounts of available DNA, global DNA methylation analysis could not be performed for patients C, D, E and G.

**Table 1:** List of analysed patients with AHCY deficiency.

Patient	Gender	Age at blood withdrawal for DNA extraction	Age at begin of dietary treatment	*AHCY* mutations	Elevation of plasma SAH levels	Elevation of plasma SAM levels	SAM/SAH ratio	References
A	m	~ 1 year	> 1 year	c.428A>G; p.Y143C (pat)c.336G>A; p.W112X (mat)	150x	30x	0.59	[5,6]
B	m	After birth	< 0.5 years	c.428A>G; p.Y143C (pat) c.336G>A; p.W112X (mat)	5x	1,6x	1.13	[6]
C	m	After birth	< 0.5 years	c.428A>G; p.Y143C (pat) c.336G>A; p.W112X (mat)	6,5x	1,8x	0.28	[10]
D	f	~ 1 month	< 0.5 years	c.428A>G; p.Y143C (pat) c.336G>A; p.W112X (mat)	unknown	unknown	unknown	unpublished
E	m	~ 26 years	1–6 years	c.428A>G; p.Y143C (pat) c.266C>T; p.A89V (*de novo*)	28x^a^	20x^a^	0.71	[7]
F^b^	f	~ 14 days	76 days	c.145C>T; p.R49C (pat) c.257A>G; p.D86G (mat)	81	23	0.28	[8, 11]
G	f	> 1,5 years	22 months	c.982T>G; p.Y328D (pat) c.428A>G; p.Y143C (mat)	200x	50x	0.62	[21]

Patients A-D are siblings. Treatment by dietary methionine restriction and supplementation with creatine and phosphatidylcholine lead to normalisation of SAH levels [5, 6]. Dietary treatment for patient E was stopped after 5 years and included restriction of methionine to 20 mg/kg/day and of protein to 1.0–1.4 g/kg/day. Patient F was dietary treated with 50% methionine-free formula (Hominex®) and 50% Pregestimil®, providing 27 mg/kg per day of methionine, and supplements of creatine (3 g/day) and phosphatidylcholine (1,200 mg/day) [8]. Dietary therapy of Patient G consisted of methionine restriction (≤ 35 mg/kg/day; i.e. ≤ 2 g total dietary protein per kg/day) and dietary supplements of creatine (300 mg/kg/day) and phosphatidylcholine (200 mg/kg/day) [21]. SAM/SAH ratios were directly taken from the references or calculated with the absolute concentrations given in the references. m = male, f = female, pat = paternal, mat = maternal

^a^ values obtained at age of 26 years

^b^ died at age of 4 months

### Methylation analysis of imprinted gene DMRs as well as LINE1 and Alu repetitive elements using bisulfite pyrosequencing

**Fig 2:**
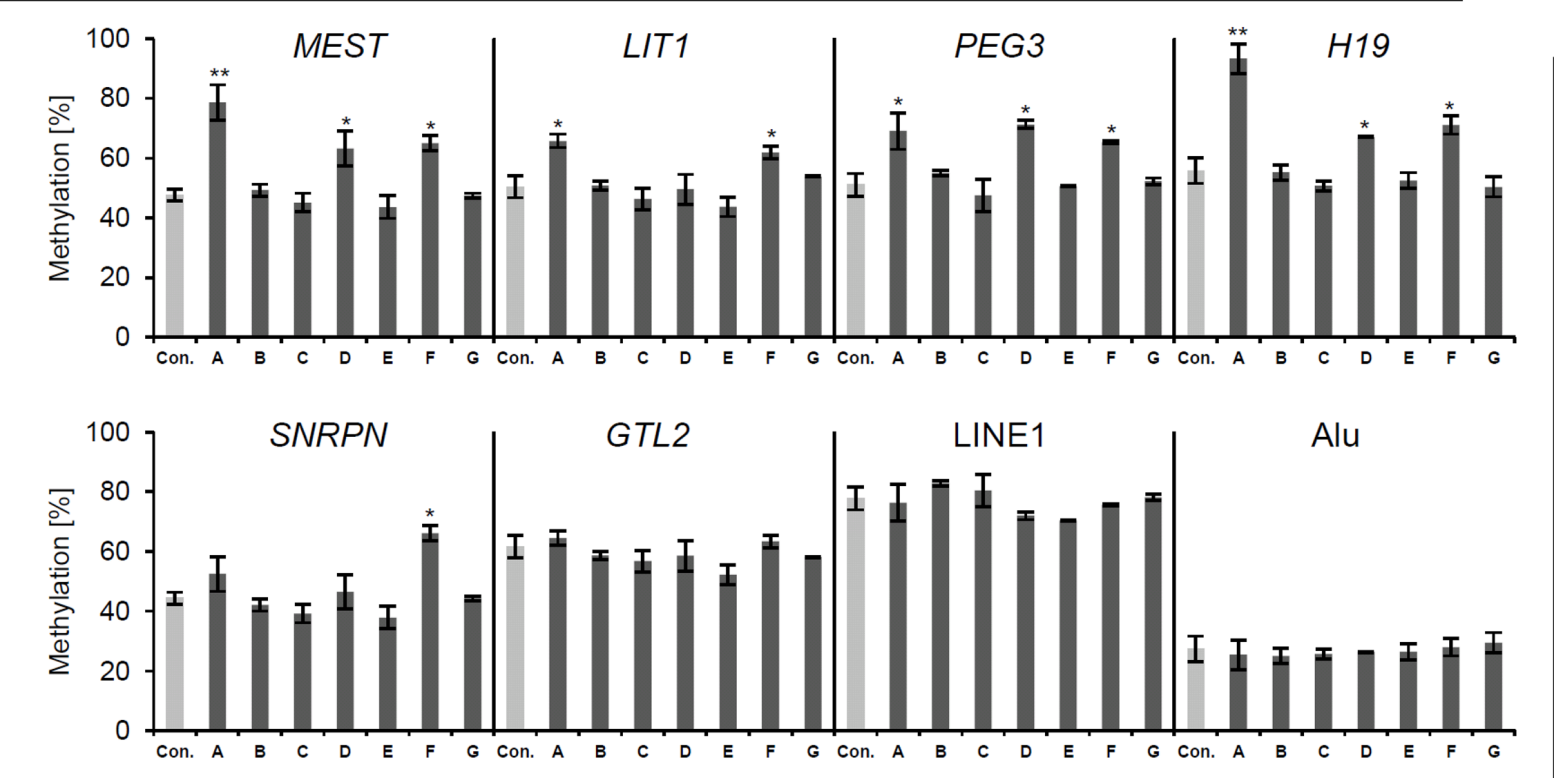
Three patients with AHCY deficiency show abnormal hypermethylation in some imprinted gene DMRs. Bisulfite pyrosequencing results of six imprinted gene DMRs as well as LINE1 and Alu repetitive elements in seven patients and controls. Only patients A, D and F showed abnormal hypermethylation of some, but not all imprinted gene DMRs. No methylation errors were observed for the repetitive elements. The bars show the mean methylation values, the error bars display the standard deviation of two to four independent measurements. Moderately abnormal methylation values are indicated by a single asterisk (*), highly abnormal methylation values by a double asterisk (**).

Using bisulfite pyrosequencing, we quantified the methylation levels of six imprinted gene DMRs (*MEST*, *SNRPN*, *LIT1*, *H19*, *GTL2* and *PEG3*) as well as LINE1 and Alu repetitive elements (Table 2 and Fig 2) in all seven patients (Table 1). Methylation was regarded as moderately or highly abnormal (hyper- or hypomethylated) if the measured methylation values were at least 10% or 20% higher or lower than the mean methylation values measured in the control individuals. For all analysed regions, the threshold of 10% represented more than twice the standard deviation (SD) of methylation values determined in control individuals.

**Table 2:** Methylation levels determined by bisulfite pyrosequencing analysis.

Patient	*MEST* (5 CpGs)	*LIT1* (2 CpGs)	*PEG3* (3 CpGs)	*H19* (3 CpGs)	*SNRPN* (6 CpGs)	*GTL2* (5 CpGs)	LINE1 (3 CpGs)	ALU (3 CpGs)
**A**	**78,64 ± 5,86**	**65,76 ± 2,29**	**69,11 ± 6,13**	**93,33 ± 4,99**	52,46 ± 5,01	64,47 ± 0,56	76,33 ± 2,79	25,45 ± 1,77
**B**	49,31 ± 1,95	50,81 ± 1,42	54,95 ± 0,85	55,24 ± 2,55	42,03 ± 4,73	58,68 ± 2,56	82,84 ± 5,05	25,05 ± 1,54
**C**	45,16 ± 3,04	46,29 ± 3,67	47,61 ± 5,36	50,69 ± 1,67	39,16 ± 3,76	56,74 ± 2,03	80,41 ± 11,13	25,75 ± 1,49
**D**	**63,24 ± 5,79**	49,60 ± 5,04	**71,25 ± 1,32**	**67,00 ± 0,24**	46,43 ± 5,07	58,57 ± 2,22	71,98 ± 1,32	26,34 ± 1,27
**E**	43,62 ± 3,80	43,66 ± 3,28	50,57 ± 0,24	52,53 ± 2,61	37,88 ± 6,89	52,24 ± 2,61	70,44 ± 1,50	26,34 ± 1,81
**F**	**65,08 ± 2,46**	**61,87 ± 2,12**	**65,29 ± 0,42**	**71,12 ± 3,01**	**66,08 ± 3,97**	63,36 ± 1,36	75,60 ± 1,77	27,98 ± 0,53
**G**	47,48 ± 0,84	53,81 ± 0,24	52,29 ± 1,11	50,38 ± 3,36	44,32 ± 3,46	58,11 ± 0,98	78,08 ± 1,51	29,49 ± 0,03
**Controls (n = 8)**	47,58 ± 1,99	50,49 ± 3,72	51,09 ± 3,82	55,85 ± 4,25	44,37 ± 1,87	61,59 ± 3,57	77,80 ± 2,27	27,36 ± 1,00

Methylation levels [%] of six imprinted gene DMRs and two repetitive elements were measured. The number of analysed CpG sites is given in brackets below each analysed region. Samples with methylation levels differing by more than 10% to those of controls are highlighted in bold, samples with methylation levels differing by more than 20% are highlighted in bold and underlined.

We detected abnormal methylation values in three of the seven analysed patients. All cases of abnormal methylation values were associated with increased methylation, i.e. we only observed abnormal hypermethylation and no abnormal hypomethylation in the three patients. Patient A was the only patient that displayed highly abnormal hypermethylation that affected *MEST* and *H19*. In addition, patient A showed moderately abnormal hypermethylation for *LIT1* and *PEG3*. Patients D and F exhibited moderately abnormal hypermethylation for *MEST*, *PEG3* and *H19*. Patient F further displayed moderately abnormal hypermethylation for *LIT1* and *SNRPN*. We found no abnormal methylation values for *GTL2* as well as the LINE1 and Alu elements.

In addition, we did not find a correlation between the elevation of SAH levels (Table 1) as a reference for severity of AHCY deficiency and DNA methylation changes. Patient G exhibited the highest elevation of SAH levels (200-fold), but no abnormalities in DNA methylation.

**Fig 3:**
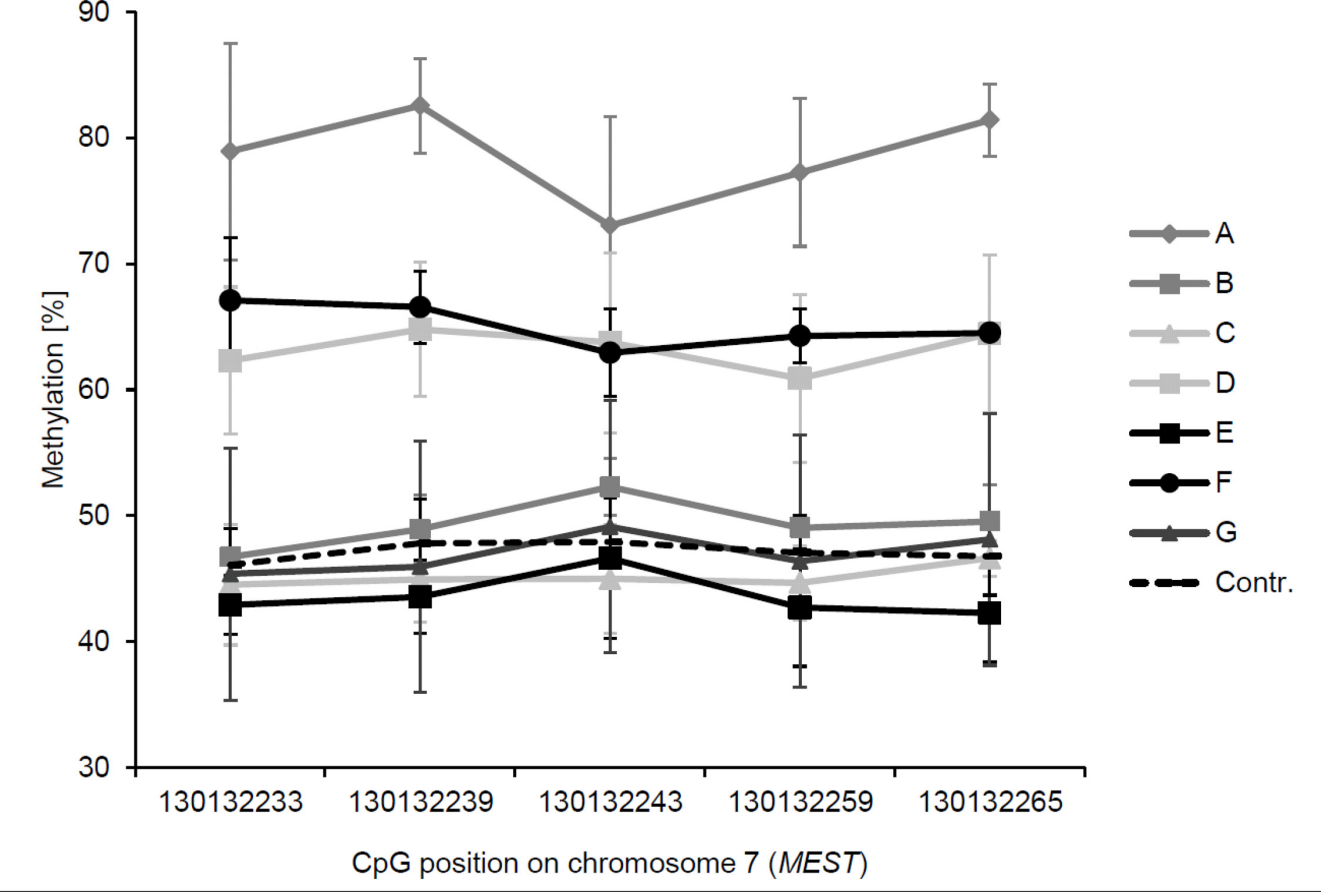
Methylation values of single CpG sites analysed in the *MEST* bisulfite pyrosequencing assay. DNA methylation [%] determined by pyrosequencing at five consecutive CpG sites in the *MEST* DMR in the seven patients and controls**.** The CpG sites within the pyrosequencing assay are ordered according to their indicated position in the genome (Ensembl version 76).

To analyse if AHCY deficiency only stochastically affects the methylation of single CpG sites in the analysed regions, we also compared the methylation values of all CpG sites included in the same pyrosequencing assay. Methylation values of CpG sites included in the same assay did not differ by more than 10% in any analysed gene and sample (exemplarily shown for *MEST* in Fig 3). These results indicate that hypermethylation of imprinted genes induced by AHCY deficiency generally affected all and not only single CpG sites included in the analyzed DMRs.

### Verification of pyrosequencing data with high resolution melting analysis

**Fig 4:**
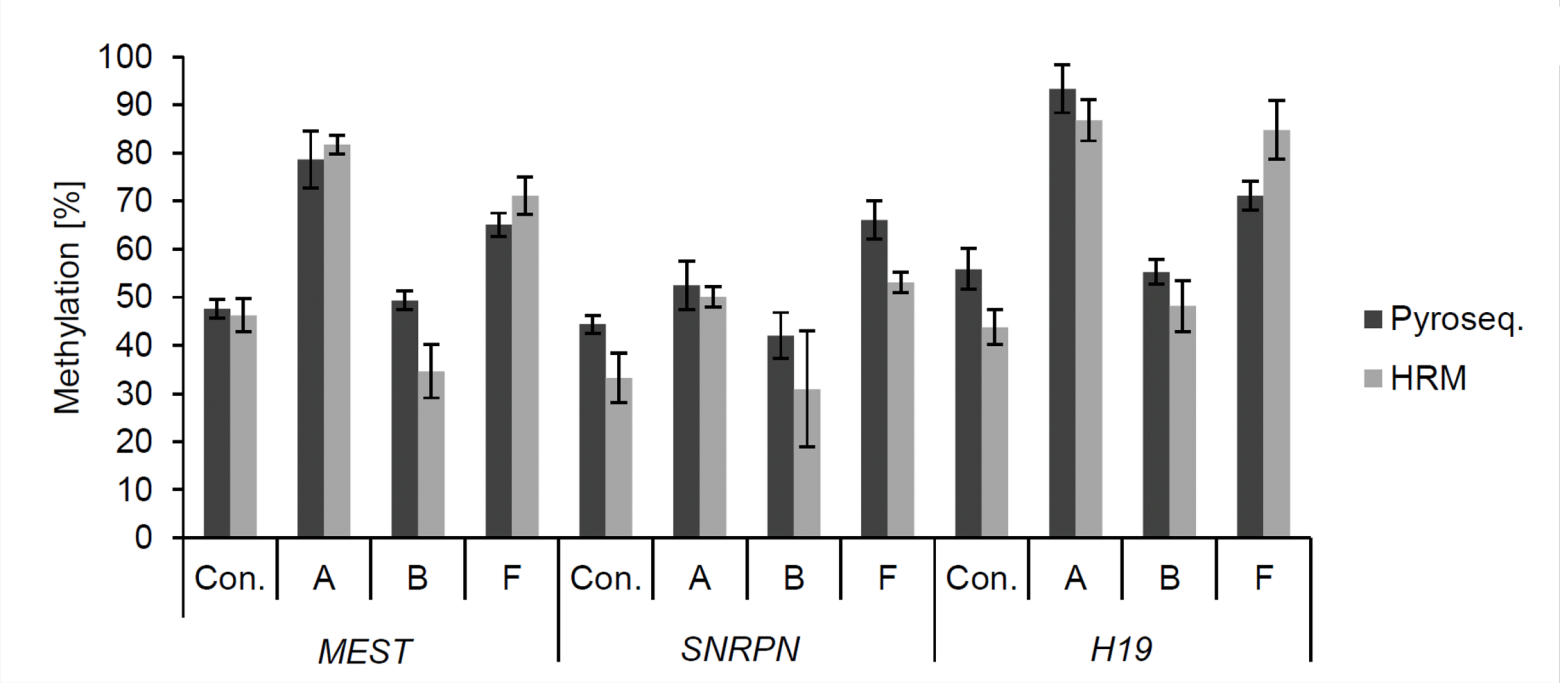
HRM analysis confirms pyrosequencing data of patients A, B and F. High resolution melting (HRM) analysis gives the mean methylation status of the complete PCR amplicons of three imprinted gene DMRs. PCR primers for the *MEST*, *SNRPN* and *H19* DMRs used for pyrosequencing also worked for HRM analysis to confirm pyrosequencing results. DNA of patients A, B and F were analysed together with controls. The mean values ± standard deviation did not differ by more than 10% methylation between pyrosequencing and HRM analysis for any of the analysed DMRs. The bars show the mean methylation values, the error bars the standard deviation of two independent measurements.

To confirm the results of the bisulfite pyrosequencing analysis, we additionally used high resolution melting (HRM) analysis to quantify the methylation of *MEST*, *SNRPN* and *H19* in patients A, B and F as well as three controls. HRM analysis quantifies overall methylation status of the whole PCR amplicon (i.e. mean methylation of all CpG sites of the PCR product), but does not allow to measure a methylation value for a single CpG site. We detected mean methylation values ± SD (standard deviation) for the three analysed imprinted genes that did not differ by more than 10% compared to the mean methylation values determined by bisulfite pyrosequencing (Table 3 and Fig 4). Thus, we were able to confirm the moderately and highly abnormal hypermethylation found for *MEST* and *H19*, respectively, in patients A and F as well as the moderately abnormal hypermethylation established for *SNRPN* in patient F.

**Table 3:** Methylation levels determined by high resolution melting analysis.

Patient	*MEST* (23 CpGs)	*H19* (18 CpGs)	*SNRPN* (21 CpGs)
**A**	81,76 ± 1,85	86,84 ±4,31	50,07 ± 2,07
**B**	34,62 ± 5,51	48,14 ±5,38	30,95 ± 11,96
**F**	71,12 ± 3,85	84,78 ±6,05	53,07 ± 2,07
**Controls**	46,24 ± 3,49	43,74 ±3,68	33,26 ± 5,21

Methylation levels [%] of three imprinted gene DMRs were measured. The number of analysed CpG sites is given in brackets below each analysed region.

## Discussion

In the present study, we have shown abnormally increased global DNA methylation in two (patients A and B) of three tested patients with AHCY deficiency and abnormal hypermethylation of imprinted gene DMRs in three (patients A, D and F) of seven tested patients with AHCY deficiency. For the two patients A and B displaying abnormal hypermethylation at the global level these observations confirm the increased global DNA methylation already reported in a previous study [5, 6]. Our finding of DNA hypermethylation in overall four (patients A and B with global hypermethylation and patients A, D and F with imprinted gene hypermethylation) but not in the remaining three (patients C, E and G) analysed patients with AHCY deficiency suggests that abnormally increased methylation is a frequent but not a constant feature associated with AHCY deficiency.

Global DNA methylation changes have also been reported for other monogenic syndromes. To our knowledge, however, patients affected by these syndromes never exhibited DNA hypermethylation, but DNA hypomethylation or a mixture of DNA hypomethylation and hypermethylation signatures. One prominent example is the rare autosomal recessive Immunodeficiency, Centromeric Instability, Facial Anomalies (ICF) syndrome which, in about 60% of cases, is caused by mutations in the gene encoding the *de novo* DNA methyltransferase DNMT3B [22, 23]. Patients with ICF syndrome have a constitutional methylation defect with severe hypomethylation mainly affecting heterochromatic sequences [24]. Other examples are the Hutchinson-Gilford Progeria (HGP) and Werner syndrome (WS), two premature aging disorders characterized by common natural aging already in childhood and attributed to mutations in the lamin A (*LMNA*) and the Werner syndrome RecQ helicase like (*WRN)* genes [25–27]. Array-based genome wide DNA methylation profiling of HGP and WS patients with either causative mutations in the *LMNA* and *WRN* genes or unknown etiology revealed aberrant DNA methylation and global DNA methylation differences with DNA hypomethylation and hypermethylation signatures between *LMNA* mutant, *WRN* mutant and non-mutant patients that also affect genes involved in aging processes [28]. Interestingly, a connection between the molecular pathogenesis of HGP and modulations of the folate–homocysteine–methionine axis has been already suggested but up to now not experimentally proven [29].

Our results must be further discussed in the light of the current knowledge on the existence of an imprinted gene network (IGN) that exerts a critical role in regulating embryonic growth [30]. The *H19* gene was suggested to represent an important trans regulator in the fine-tuning of this IGN in the mouse embryo by controlling the expression of several other imprinted genes including *Igf2*, *Slc38a4*, *Dcn*, *Dlk1*, *Peg1/Mest*, *Gtl2*, *Cdkn1c* and *Igf2r* [31]. Further studies in the mouse showed that a complex between the *H19* long noncoding RNA and the methyl-CpG–binding domain protein 1 MBD1 is needed for the control of five of these genes (*Igf2*, *Slc38a4*, *Dcn*, *Dlk1*, and *Peg1/Mest*) with MBD1 binding directly to the DMRs of three of them (*Igf2*, *Slc38a4* and *Peg1/Mest*) [32]. Interestingly, both *H19* and *MEST* displayed abnormally increased methylation levels in the three AHCY patients for whom hypermethylation of several imprinted genes was detected out of the seven patients analyzed. In addition, *H19* and *MEST* even were the only two imprinted genes for which highly abnormal hypermethylation was detected in one of these three patients (patient A). Thus, it is tempting to speculate that some patients with AHCY suffer from a general deregulation of the IGN due to abnormal hypermethylation of its master regulator *H19*. As a limitation of this hypothesis, it has, however, also to be considered that *GTL2* as another IGN member regulated by *H19* is not affected by abnormal hypermethylation at all.

Only one previous study that analysed mice with diet-induced hyperhomocysteinemia reported an association between increased SAH levels and abnormal gene-specific hypermethylation affecting the *Fads2* gene (encoding Δ(6)-desaturase) [33]. Other human and mouse studies analysed healthy young women, *Mthfr* (methylenetetrahydrofolate reductase) knockout mice and *Cbs* (cystathionine β-synthase) heterozygous knockout and wild-type mice fed a control or methyl-deficient diet, respectively. These studies exclusively correlated the elevation of SAH with decreased DNA methylation [34–36]. Further reports on Sprague-Dawley rats and *ApoE* knockout mice with induced hyperhomocysteinemia and 2 to 6-fold elevated SAH levels specifically associated the DNA hypomethylation with repetitive B1 elements, the murine equivalent of human Alu repetitive elements [37, 38].

In AHCY deficient patients, however, LINE1 and Alu elements did not show abnormal DNA methylation. This is not completely unexpected since particularly LINE1 methylation has already been reported to exhibit little variability in blood [39] and thus has been questioned to be sensitive enough for detecting subtle changes in DNA methylation. In addition, bisulfite pyrosequencing of LINE1 and ALU elements uses consensus sequences to determine methylation levels of only 3 to 4 CpG sites among half a million of LINE‐1 and ∼1.4 million Alu elements [40]. Thus, the number of assessed elements is unclear and may even vary between individuals making the assays not entirely representative of global DNA methylation [41]. Further limitations of these assays with regard to the representation of global DNA methylation could arise from the fact that methylation of repetitive elements may be regulated by different mechanisms than methylation of the other parts of the genome [42].

In all studies mentioned above, DNA hypomethylation was attributed to the action of SAH as a potent product inhibitor of SAM-dependent methyltransferases [43]. In the light of these data, our finding of DNA hypermethylation in AHCY deficient patients seems to be rather unexpected. However, the mentioned studies also showed a decrease of SAM levels, whereas AHCY deficiency increases SAM levels and therefore the amount of substrate for methyltransferases (MTs). Furthermore, AHCY deficiency leads to a much more pronounced, more than 100-fold increase of SAH [44] compared to the moderate SAH increase associated with DNA hypomethylation in the above mentioned studies. Thus, a reversal of the effect on DNA methylation may be induced by the transition from moderately elevated to highly elevated SAH levels. A similar reversal was already reported for the effect of elevated homocysteine on *CCNA1* (cyclin A) transcription: Elevation of homocysteine in clinically relevant concentrations lead to inhibited vascular endothelial cell growth via hypomethylation and transcriptional inhibition of the *CCNA1* gene that was accompanied by accumulation of SAH [45]. In contrast, elevation of homocysteine in supraphysiological concentrations promoted proliferation and transcription of *CCNA1* in vascular smooth muscle cells [46]. Hence, it can be speculated that the massive and simultaneous accumulation of SAH and SAM typically seen in AHCY deficiency may reduce or abolish product inhibition of SAM-dependent DNA methyltransferases (DNMTs).

In this context, it has further to be considered that DNMT1 was already reported to be less sensitive to SAH than other SAM-dependent non-DNA MTs [47]. In mammals, more than 200 SAM-dependent MTs have been identified [48–50]. Thus, it can be hypothesised that the non-DNA MTs may remain inhibited at higher SAH concentrations present in AHCY deficiency and leave the excess SAM to the DNMTs which then increase DNA methylation in patients. Barić *et al*. already suggested the myelin basic protein-arginine N-methyltransferase PRMT7 as one non-DNA MT that may be involved in the etiology of AHCY deficiency [51]. This enzyme catalyzes the arginine methylation of myelin basic protein, an important component of myelination, which is known to be delayed in AHCY deficiency [51]. 100-fold elevation of SAH was shown to reduce the activity of this MT by about 90% [52]. By comparison, hyperhomocysteinemia associated with increased SAH levels reduced the activity of DNMT1 only by 30% [45].

In the light of our data, three further critical questions have to be asked: (i) Why did only four of the seven patients with AHCY deficiency exhibit abnormally increased methylation values? (ii) Why were there two patients that either displayed abnormal global hypermethylation but not increased imprinted gene methylation (patient B) or increased imprinted gene methylation but not abnormal global hypermethylation (patient F)? (iii) Why did we not observe a correlation between the elevation of SAH levels as a reference for severity of AHCY deficiency and the occurrence of DNA methylation changes? Based on the hypothesis already mentioned above, it is possible that only the patients that exceeded a critical threshold of elevated SAH levels at the embryonic stage underwent fetal programming during early to mid-gestation leading to DNA hypermethylation to different degrees, i.e. not necessarily detectable at the global level, and at different genomic locations. As an example of fetal programming, supplementation of viable yellow agouti (*A*^*vy*^) mice with dietary methyl donors and cofactors involved in SAM-substrated methylation such as folate, methionine, choline, and vitamin B_12_ throughout gestation and lactation was already shown to increase methylation at the *A*^*vy*^ metastable epiallele of the agouti coat colour locus in the offspring thereby changing their phenotype from yellow obese to pseudoagouti lean [53]. The window of susceptibility for altering the epigenetic state of the *A*^*vy*^ allele and the phenotype was determined to be from early to mid-gestation [54, 55]. It is important to note that mouse embryos harbouring a complete deletion of the *Ahcy* gene die two to three days after *Ahcy* is first expressed at the peri-implantation stage which lies in the middle of the above mentioned window of susceptibility [4]. A further mouse metastable epiallele with findings similar to those reported for the *A*^*vy*^ allele is the axin fused (*Axin*^*Fu*^) locus [56]. Putative human metastable epialleles have been also already described [57] and may be promising candidates for more detailed methylation analysis in AHCY deficient patients. A global DNA methylation analysis of the patients which, owing to lack of sufficient genomic DNA, were up to now only studied for imprinted gene methylation, would be necessary to further corroborate the hypothesis of fetal programming dependent on SAH levels present around implantation. In addition, the fact that only some patients with AHCY deficiency display DNA hypermethylation and these patients are even not all affected at the global or gene-specific level may reflect the involvement of the AHCY protein in a multitude of cellular pathways and complex protein interaction networks including more than 200 SAM-dependent MTs and thereby adjusting the metabolic machinery of the cell and individual. This is further mirrored by the large variety of clinical disease presentations such as potential lethality in early life [11] and association with hepatocellular carcinoma [58] as well as by the highly diverse successful treatment strategies discussed below.

The standard therapeutic treatment of AHCY deficiency consists of a methionine-restricting diet and supplementation with creatine and phosphatidylcholine [5, 6]. This can lead to an improved development and normalisation of SAH levels [6]. Interestingly, rats that displayed a reduced SAM/SAH ratio and global DNA hypomethylation due to a 9-week long methyl-deficient diet reacquired normal global methylation values by subsequent refeeding with a normal diet. Normal global methylation levels, however, could not be restored when the rats received the methyl-deficient diet longer than 9 weeks [59]. In the light of these findings, it would be interesting to study the methylation levels of the patients again after months or even years of treatment and verify if a normalisation of methylation levels can be achieved. Barić *et al*. (2005) already analysed global DNA methylation of leukocyte DNA in patients A and B after 31 and 10 months of dietary treatment, respectively. Although methylation levels did not normalise, the authors noted a possible trend towards a reduction of global DNA methylation [6].

Another recently reported innovative therapeutic approach included the transplantation of a liver segment from a healthy, unrelated living donor to patient G and lead to a normalisation of blood methionine and SAM levels as well as an improvement of development with a 4-fold accelerated head growth and promising gains in gross motor, language, and social skills [21]. Should this therapeutic approach become available for other AHCY-deficient patients with DNA methylation changes, analysis before and after treatment could also give further insights into the possible plasticity of the methylation defects.

## Conclusions

To our knowledge, AHCY deficiency is the first monogenic disease with DNA hypermethylation that occurs as a frequent but not a constant feature and affects different genomic regions to different degrees. The massive and simultaneous accumulation of SAH and SAM typically associated with AHCY deficiency may reduce or abolish product inhibition of SAM-dependent DNA methyltransferases but not SAM-dependent non-DNA methyltransferases and thus increase DNA methylation in patients. These abnormal DNA methylation reactions may have occurred only in patients that exceeded a critical threshold of elevated SAH levels at the embryonic stage and thus underwent fetal programming during early to mid-gestation. We consider AHCY deficiency to be an ideal model disease for studying the molecular origins and biological consequences of DNA hypermethylation due to impaired cellular methylation status. A deeper insight into this disease may contribute to a better understanding of the epigenome´s plasticity and its susceptibility to critical changes in one-carbon metabolites. Specifically, further studies are warranted that monitor long-term changes in DNA methylation but possibly also histone methylation at the gene-specific and genome wide level during dietary treatment or after liver transplantation therapy.

## Materials and Methods

### Patients and controls

Patients analysed in this study are listed in Table 1. This study was performed on DNA extracted from peripheral blood samples. Due to insufficient amount of available material from some patients, not all samples could be analysed with all methods. The entire study was approved by the ethical commission of the Medical Faculty Zagreb. The informed consent for using the patients´ samples for research to their benefit has been obtained directly from the proband or biological parent in the case of minors and has been signed in the presence of the responsible physician. The ethical commission of the Medical Faculty Zagreb has evaluated the informed consent for patients A-F and has issued an approval form to the responsible scientist (OV) for usage of patient cell lines for research purposes only. The Institutional Review Board of Lancaster General Hospital approved the study of patient G and the informed consent of patient G's parents. Age- and sex-matched controls as well as adult controls were included in the study and did not show any significant methylation differences with the methods used.

### Genome wide methylation analysis

The genome wide methylation analysis of patients A, B and F as well as three control samples was performed in three technical replicates using the MethylFlash Methylated DNA Quantification Kit (Epigentek) according to the manufacturer’s instructions with 200 ng of genomic DNA for each sample. Global DNA methylation levels of patient samples were normalised to the average of the control group in three independent measurements. Variation between controls was included via error propagation. Statistical analysis was performed using Kolmogorov–Smirnov test followed by independent samples *t*-test. In a subsequent experiment, 10 further age- and sex-matched control samples (5 female and 5 male) were analysed in the same way together with the DNA of patient B to confirm that the three control samples of the first experiment are representative for the normal population.

### DNA isolation, bisulfite conversion, PCR and pyrosequencing

Human genomic DNA was isolated with the DNeasy blood and tissue kit (Qiagen) according to the manufacturer’s instructions and quantified using a NanoDrop 2000 spectrophotometer. 450 ng DNA per sample were used for bisulfite conversion with the Epitect 96 bisulfite kit (Qiagen). PCR and pyrosequencing with primers for the DMRs of *SNRPN*, *LIT*, *PEG3*, *H19*, *MEST* and *GTL2* as well as LINE1 and Alu elements were performed as described elsewhere [60]. Pyrosequencing was performed on a PyroMark Q96 ID (Qiagen) with PyroMark Gold Q96 reagents (Qiagen). Data were analysed using the PyroMark CpG Software 1.0.11 (Qiagen).

### High resolution melting analysis

HRM analyses were performed using a CFX96 Real-Time PCR Detection System (BioRad). Each PCR was performed in a final volume of 15 μl, containing 7.5 μl Precision Melt Supermix (BioRad), 400 nM of each primer, and 20 ng of bisulfite-converted DNA (theoretical concentration presuming no loss of DNA during bisulfite modification). PCR amplification was performed with one step of 95°C for 2 min; 45 cycles of 95°C for 10 s, 56/58°C for 30 s and 72°C for 15 s; followed by an HRM step of 95°C for 30 s, 60°C for 1 min, 65°C for 10 s and continuous acquisition to 90°C at one acquisition per 0.2°C. Primers for *SNRPN*, *H19* and *MEST* were the same as for bisulfite pyrosequencing PCR reactions. Buccal swab DNA from a healthy donor was used to generate fully methylated and unmethylated bisulfite-converted DNA standards. 50 ng of DNA was used for whole genome amplification using the REPLI-g midi kit (Qiagen) to generate the unmethylated standard DNA. The reaction was performed according to the manufacturer's instructions. An aliquot of 100 μg was in vitro methylated with 400 U SssI methylase and 640 μM SAM (NEB) according to the manufacturer's instructions. After 4 hours at 37°C, additional SAM and 50 units of M.SssI methylase were added and incubated overnight at 37°C to ensure complete methylation. Both methylated and unmethylated DNA samples were purified by phenol-chloroform extraction followed by ethanol precipitation and suspended in DNase-free water. They were mixed to obtain methylation levels of 0%, 25%, 50%, 75% and 100% and were included in duplicates in each assay. Commercially available bisulfite-converted DNA standards (Qiagen) were analysed together with the in house-made DNA standards and confirmed their methylation levels. HRM data were analysed using Bio-Rad Precision Melt Analysis Software (BioRad) with output plots produced as normalised melting curves. Normalised relative fluorescent units (RFUs) were exported to Prism 6 (Graphpad). Area under the curve (AUC) was calculated and the quadratic regression was used to interpolate the unknown samples to the standards (R^2^ was > 0.98). Due to the variability of the dynamic range between the standards, this method is suitable for methylation analysis of imprinted genes but not repetitive elements.

## Supporting Information

S1 Fig Genome wide methylation levels of 10 further age- and sex-matched control samples analysed together with patient B. The bars display the genome wide 5-methyl-cytosine methylation levels [%] obtained in one measurement of patient B and the mean 5-methyl-cytosine methylation and standard deviation [%] detected in one measurement of 5 female and 5 male control samples, respectively.
(EPS)
